# How Early Experience Shapes Human Development: The Case of Psychosocial Deprivation

**DOI:** 10.1155/2019/1676285

**Published:** 2019-01-15

**Authors:** Charles A. Nelson, Charles H. Zeanah, Nathan A. Fox

**Affiliations:** ^1^Boston Children's Hospital and Harvard Medical School, USA; ^2^Harvard Graduate School of Education, USA; ^3^Department of Psychiatry and Behavioral Sciences, Tulane University School of Medicine, USA; ^4^Department of Human Development and Quantitative Methodology, University of Maryland, USA

## Abstract

Experience plays an essential role in building brain architecture after birth. The question we address in this paper is what happens to brain and behavior when a young child is deprived of key experiences during critical periods of brain development. We focus in particular on the consequences of institutional rearing, with implication for the tens of millions of children around the world who from an early age experience profound psychosocial deprivation. Evidence is clear that deprivation can lead to a host of both short- and long-term consequences, including perturbations in brain structure and function, changes at cellular and molecular levels, and a plethora of psychological and behavioral impairments.

## 1. Introduction

Experience is the engine that drives much of postnatal brain development. Based primarily on research using rodent and nonhuman primates, a great deal is known about how the nature and the timing of experience influences the course of the developing brain. Not surprisingly, the *absence* of key experiences during these critical periods can exert serious and in some cases, lasting effects on multiple domains of development. For example, much has been learned from studies of rodents and nonhuman primates in which sensory loss is induced (e.g., the animal is deprived of light or sound; [1]) or in which animals are selectively reared (e.g., deprived of seeing faces; [2]). Similarly, great insight into how the absence of experience alters brain development has been gained by studying human infants who have experienced sensory loss early in life, such as those born with cataracts or who are born deaf, and who subsequently have their vision or hearing restored at different points in development [3–6].

A far more insidious and widespread form of deprivation involves the millions of children around the world who experience psychological neglect early in life—for example, children who are neglected by their families (>500,000 in the US alone in 2013; [7]), children left behind by parents who have migrated to another country to look for work (61 million in China in 2014; [8]), or children who are orphaned or abandoned by their parents and then reared in institutions (>140 million abandoned/orphaned children, 8 million living in institutions; [9, 10]).

Here, we discuss how early psychosocial deprivation during critical periods of development shapes neural, biological, and behavioral development during childhood and beyond. Drawing from research on rodents, nonhuman primates, and humans, we consider what is known about the timing of deprivation as well as the timing of recovery from deprivation—specifically, whether critical periods impose constraints on recovery. We begin our critical and selective review of findings related to critical periods by discussing what is known in development, drawing first on the animal literature and then turn our attention to the literature on human infants. We then consider one specific type of experience that is common across rodents and mammals, that is, maternal caregiving. We review what is known about the influence of timing of caregiving on species-typical socioemotional development. We then summarize the findings from the Bucharest Early Intervention Project, the only project of its kind to examine the effects of early intervention in a randomized control trial with children who have been abandoned and are living in institutions. We specifically highlight findings that address the issue of critical periods in human development during which the influence of experience has a significant impact for particular domains. We conclude our review by discussing the implications this knowledge has for the millions of children around the world who experience inadequate caregiving because they have been abandoned, orphaned, or raised in a neglecting family.

## 2. Conceptual Framework: Critical vs. Sensitive Periods

A key issue in modeling the effects of inadequate caregiving on development is to understand the issue of *timing* of exposure to adversity and timing of environmental enhancement—this concept of timing is generally referred to as a sensitive or critical period. Although “sensitive periods” and “critical periods” are often used interchangeably, they differ in fundamental ways. Knudsen [11], for example, has argued that *sensitive period* is a broad term often used to describe the effects experience has on the brain during limited periods in development. If a key experience fails to occur during a sensitive period, it may be difficult, without tremendous effort, to redirect development along a typical trajectory; even then, function in the affected domain (e.g., language) may not fully recover. A human infant forming a secure attachment to a caregiver seems to reflect a sensitive period. *Critical periods*, by contrast, result in irreversible changes in brain function. If a key experience fails to occur during a critical period, behavior is believed to be permanently affected. Filial imprinting in animals likely represents a critical period.

**Figure 1:**
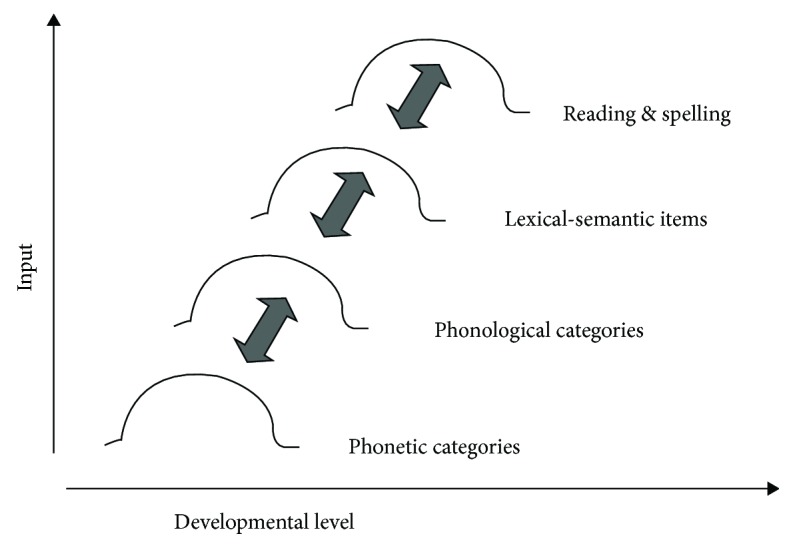
Possible multiple sensitive periods for the speech processing system. Figure was reproduced from Werker and Tees [13] (under the Creative Commons Attribution License/public domain).

Of course, both sensitive and critical periods represent time windows during which experience exerts a particularly strong influence on neural circuit formation. Knudsen [11] has argued that whatever plasticity exists *beyond* a sensitive period is constrained by what transpired *during* a sensitive period. In other words, one can reshape existing circuits only to a limited degree. Two additional points are also worth noting. First, there are cascades of sensitive/critical periods during development; thus, there will be multiple, cascading critical periods for different neural circuits and for different complex phenomena such as caregiving and language. Moreover, even *within* a domain there will be different critical periods (for example, within the domain of language, there may be different critical periods for language discrimination, understanding word forms, and for discriminating phonological categories; [12]). An example of this may be the conceptual model presented by Werker and Tees [13]. See Figure 1.

**Figure 2:**
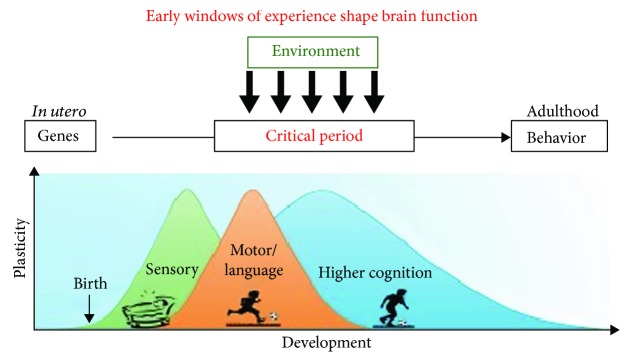
Figure illustrating the interaction of experience and maturation during critical periods in development. Figure was reproduced from Hensch and Bilimoria [16] (under the Creative Commons Attribution License/public domain).

Second, great inroads have recently been made in understanding the molecular cues and brakes that regulate critical periods, including how to lift such brakes [14, 15]. Because the term “critical period” has endured in the popular lexicon, we use that term throughout this paper, although in nearly all instances the phenomena we describe most likely reflect sensitive periods.  Figure 2 [16] illustrates the concept of critical periods. The X axis of this figure represents age and development, and the Y axis represents degree of neural plasticity. There are multiple factors presented in this figure. First, as can be seen, there are the contributions early in life of genes that program brain development. Second, as can be seen different domains (sensory, language, cognitive) have different trajectories of increasing and then diminishing plasticity across development, suggesting different times when experience for these different domains will have its most profound impact. Finally, the figure suggests that there are windows of plasticity or critical periods across these different domains of functioning.

## 3. Animal Models of Psychosocial Deprivation and Inadequate Caregiving

A variety of data from rodents and nonhuman primates have addressed issues regarding critical periods in the development of typical and atypical behavior. These studies have manipulated the timing of early experience and the quality of caregiving in the development of neural and physiological systems that support physical growth, stress responsivity, and homeostasis. Although detailed molecular mechanisms involved in each of these aspects are still under study, there is a convergence of this work that emphasizes the importance of early experience and, particularly in the rodent, the presence of critical periods early in postnatal life during which experience plays a singular role.

### 3.1. Stress Hyporesponsive Periods in Rodents

Research with rodents provides a particularly unique opportunity to manipulate many of the variables that are important in early experience, including timing of an event and quality of that event. Some of the first work on this topic was conducted by Levine [17] and Denenberg et al. [18], in which the precise time when particular types of postnatal experience occurred was manipulated (e.g., handling of the rat pup outside the nest) and outcomes of such manipulation on stress physiology were examined. For example, Levine [19] removed rat pups from the nest at different times after birth and examined the rat's subsequent ability to mount a cortisol response in reaction to a stressor. He found that timing of removal from the nest (and handling that occurred when the rat pup was removed) affected cortisol responses. Denenberg [18] found that pups removed from the dam on the 10th day of postnatal life were affected as adults in their ability to learn and to regulate their emotion and state of arousal [20]. Pups handled in the first ten days were also better able to deal with later stressors [21]. This early work suggested that there is a stress hyporesponsive period (i.e., when the system was not responsive to external stressors). Subsequent work by Plotsky et al. [22] and by Roth and Sullivan [23] has shown that the presence of the rat dam early in life was critical in regulating the stress response of the pup. Anticipating the work that would occur almost 50 years later, Denenberg and Whimbey [24] found that rat pups that were handled at 20 days postnatal life had offspring that were more fearful than control animals (animals from rat dams that were not handled). Indeed, whether the rat dam was the biological mother or foster mother mattered less than the history of handling that the biological mother had as an infant on her infant's behavior. This work presages the epigenetic processes elucidated by Meaney and others, showing the intergenerational effects of early experience on later emotionality in offspring (e.g., [25]).

The work of Roth and Sullivan and that of Sullivan and Gratton [23, 26] are notable here as it expanded and revised the idea of a stress hyporesponsive period in the rat pup. Sullivan charted a sequence of critical periods during which, in the presence of the rat mother, the pup is hyporesponsive to stress. Indeed, if the dam is given a scent (e.g., peppermint) and the pup is fear conditioned (shocked paired with the odor) to that scent, the shock will not elicit a stress/cortisol response. Sullivan and Wilson [27] have detailed the neural structures and hormonal regulators that appear responsible for this lack of stress response. Essentially, in the early postnatal days of life of the rat pup, connections between the amygdala and prefrontal cortex are not established. Once these are established, the rat pup will mount an adult-like stress response even in the presence of its mother. Thus, the effects of early handling and early experience are a function of context (presence or absence of the mother) and appear to target brain structures (amygdala, prefrontal cortex, and hippocampus) that are integrally involved in stress physiology.

The effects of inadequate maternal care on infant development also have been examined. Denenberg et al. [18] studied the effects of having rat dams rotate between litters. His studies suggested the centrality of consistent caregiving for pup survival. In more recent work, Ivy et al. [28] have proposed a model in which they produced inadequate care in female rats by restricting the nesting materials for the dam with her pups in the cage. These restrictions led to fragmented interactions between the dam and the pups. This abnormal activity was accompanied by inadequate care—anxious-like behaviors—and by increased stress physiology, suggesting that the dams were under chronic stress. In addition, the rat dams did less licking and grooming of their infant pups than control dams. These studies examine the effects of manipulating environmental resources on maternal caregiving and provide evidence on perturbations in infant behavior as a result of problematic and inadequate maternal caregiving.

### 3.2. Maternal Caregiving Disruptions in Rodents

Other research has examined the interactions between the rat dam and her pups to identify the joint influences of each on the physiology of the other. Hofer [29], for example, separated and experimentally manipulated different aspects of the presence of the rat dam on the rat pup, including milk, body warmth, smell, and movements. He and his colleagues showed that each of these aspects of the rat dam “regulated” the physiology of the rat pup, and this in turn regulated the rat dam's physiology. His work showed that maternal proximity during a critical period of the rat pup's development operated to downregulate the rat pup's physiological functioning. His interest and emphasis on lactation anticipated the work that is now central to the hormonal bases of caregiving in the role of oxytocin [30]. Hofer [31] coined the term “hidden regulators” to describe this effect because there were no obvious behavioral referents for these regulators. Hofer's microlevel detail about the temporal synchrony between the rat dam and her pups served as an important impetus for the studies of face to face interaction in human infant-caregiver pairs [32].

### 3.3. Maternal Caregiving Disruptions in Nonhuman Primates

Animal research on the importance of maternal caregiving has not been limited to rodents. For example, the work on the effects of maternal separation was extended to nonhuman primates by Harlow and Zimmermann more than half a century ago [33]. In a series of studies, infants were separated from their mothers at an early age, and either reared in isolation or with peers. These separated (and in some instances, isolated) animals exhibited symptoms of depression and motor stereotypies. More importantly, when these animals were exposed to younger peers, this experience appeared to reverse many of the negative effects of the early separation [33]. The work of Harlow and Suomi [34] delineated how maternal deprivation and being raised with peers led to animals who were anxious and impulsive as adults and displayed an abnormal stress response [35].

Rosenblum and Paully [36] examined the effects of inadequate care in Bonnet macaques. They observed infants where the mother had either consistently available resources, lack of resources, or inconsistent/unpredictable conditions. They reported that in the inconsistent conditions infants displayed significantly greater emotionality and alternations in their stress physiology. Sanchez and colleagues [37] also demonstrated that inconsistent and abusive caregiving in the Rhesus macaques compromised infant behavior and stress physiology. Across these studies, there is strong evidence that inadequate care is associated not only with heightened stress physiology in the infant but also maladaptive behaviors as they mature.

O'Connor and Cameron as well as Sabatini et al. [38, 39] assessed the effects of maternal deprivation in Rhesus macaque infants by removing the mother from the infant's social group at different infant ages. This led to dramatic social abnormalities and aberrant behaviors in the infant monkeys depending upon whether the mother was removed at 3 months, one month, or one week after birth. The earlier the removal, the more disturbed the behaviors in the monkey. Many abnormalities in these maternally deprived monkeys persisted into adulthood, and they were associated with reductions in dendritic branching in the prefrontal cortex and in gene expression in the amygdala.

The notion of critical periods may be traced to the work of ethologists such as Konrad Lorenz who described imprinting in birds. Lorenz noted that baby ducklings would follow the individual who moved within their line of sight right after they were hatched. If there was no one there, they would not imprint. If a bird was only present after a certain period of time, then the ducklings would not imprint. A moving stimulus was most effective in initiating imprinting during the “critical period” [40].

Hubel and Wiesel's studies on the visual system reinforced the idea that experiences during a critical period impacts typical development. Hubel and Wiesel were interested in the effects of early experience on the typical development of visual function. They completed experiments first on cats, and then on monkeys, in which they either deprived a single eye or both eyes of visual experience and examined the changes that occurred in the occipital cortex, the area of the brain involved in early visual processing. They found that depriving one eye of typical visual experience led to aberrant vision, and in fact the second eye actually “took over” regions of the occipital cortex normally activated by the other eye. In addition, there appeared to be a sensitive period during which experience had a significant role in the development of typical visual processing. After that sensitive period, it was more difficult for brain organization supporting typical visual processing to occur [1].

Hubel and Wiesel's work stands as the preeminent work on the effects of early experience and sensitive periods. There have been a number of other researchers who have examined the idea of sensitive periods in the visual and other domains in human infants. Before describing that work, it is important to have a clear definition of just what a sensitive period means. Eric Knudsen, a neurobiologist at Stanford who studies the effects of early experience, writes:

“*Experience exerts a profound influence on the brain and, therefore, on behavior. When the effect of experience on the brain is particularly strong during a limited period in development, this period is referred to as a sensitive period. Such periods allow experience to instruct neural circuits to process or represent information in a way that is adaptive for the individual. When experience provides information that is essential for normal development and alters performance permanently, such sensitive periods are referred to as critical periods.*” [41]

Knudsen differentiates between a sensitive and critical period. A sensitive period is one during which experience exerts its effect during a limited time. However, when experience is essential and alters performance permanently, then such a period is called a critical one. He provides a possible mechanism by which this may occur in the brain, in which experience “instructs” neural circuits to process information. That is, somehow, experience wires brain circuitry in a way that is adaptive for the individual.

## 4. Summary

The picture that emerges from research with rodents and nonhuman primates on maternal deprivation is compelling: unless deprivation ends early, by reuniting the animal with its biological mother *or* by cross fostering the animal with another, adequate mother, there are long-term consequences of early maternal deprivation. Deprived offspring exhibit symptoms of what in the human would be considered anxiety or depression. They show cognitive deficits (e.g., poorer spatial memory, reduced interested in novelty), and more importantly, they show a variety of attachment-related problems, including indiscriminate social behavior. Similar findings are found among animals reared with mothers who provide inadequate care.

## 5. Caregiving Quality in Human Psychological Development

Human infants are born requiring the care and support of adult caregivers for survival. An essential role of parenting in the earliest years of life is providing regulation that assists the developing immature infant. Through reading and responding to infant behavioral cues, caregivers provide essential input necessary for the proper elaboration of essential domains of development, such as stress response systems, attentional systems, and attachment.

### 5.1. The Stress Response System

There is considerable development and plasticity across the first few years of life in the developing stress response systems—the hypothalamic-pituitary-adrenocortical system and the autonomic nervous system. As in studies with rodents, inadequate caregiving such as severe psychosocial neglect has been shown to disrupt concurrent and later functioning of both of these systems [41–43]. However, caregiver relationships characterized by responsive caregiving seem to buffer the young infant's cortisol responses and enhance recovery during stressful situations [44, 45]. Further, experiments assessing interventions to restore adequate caregiving and enhance parenting in the early years have demonstrated restoration of healthier diurnal cortisol regulation, cortisol responses to stress, and autonomic nervous system responses to stress [41, 46].

### 5.2. Attachment

Human infants have a propensity to form selective attachments to their caregivers by 7 to 9 postnatal months under typical circumstances. Only in extreme conditions of neglect or deprivation do human infants fail to form such attachments [47–49]. Early patterns of interaction between infants and parents are predictive of subsequent qualitative differences of attachment between them, and characteristics of parents assessed prenatally have been shown to predict individual differences in the quality of attachment between infants and parents more than one year later [50, 51]. Interventions designed to enhance caregiving quality have been shown experimentally to enhance security of attachment in high-risk groups [52–54].

Though infants contribute to coregulated patterns of interaction during the first year of life, the direction of effects in early infancy is largely parent to infant. Beginning soon after birth, caregivers adapt their behaviors by responding to newborn states of alertness, leading to synchronous interactions [55]. Through rhythmic crossmodal matching of infant behaviors, emotional states, and biological rhythms, parents shape infants' relational responses [56–58]. This biobehavioral synchrony between infants and parents provides experiences for infants that lead to healthy development of stress response systems, regulated attention, and secure attachments [56, 59]. Adverse environments that fail to provide these experiences lead to disruptions in these domains.

## 6. The Psychological and Biological Toll of Early Psychological Deprivation

Having established that access to species-typical (adequate) caregiving during critical periods of development plays an important role in subsequent psychological and neurobiological development, we now turn our attention to a more precise examination of the role of critical periods in human infants deprived of adequate caregiving in the first months and years of life. We begin this section with a brief overview of what is known about an increasingly studied model of human deprivation—the effects of institutional rearing on development. We then turn our attention to whether the negative sequelae of early institutional rearing can be reversed by removing children from institutions and providing them with adequate caregiving. A particular theme highlighted is timing—whether recovery from institutional rearing is influenced more by the duration of institutional care or by the age of placement into adequate caregiving environments. We conclude this section by highlighting some of the major unresolved issues and then close out the paper with a discussion of the scientific and policy implications of such work on psychological deprivation.

## 7. The Effects of Institutional Rearing on Development

Since the turn of the 20th century, there has been interest in the effects of institutional rearing on young children's behavior. After World War II, Bowlby wrote a report for the World Health Organization in which he described the conditions of orphaned and abandoned children living in institutions and cautioned about the negative effects of psychosocial deprivation on the cognitive and socioemotional development of the young child [60]. In the United States, Goldfarb demonstrated the negative effects of institutionalization on children's behavior with emphasis on externalizing behavior and aggression [61, 62]. Spitz also described a syndrome referred to as hospitalism that was the result of infants being left in pediatric units without appropriate social stimulation [63].

Over the years, there have been numerous studies of infants and young children growing up in institutional settings (e.g., [64, 65]). Though none of these involved randomized controlled trials, many involved comparisons among children with regard to the age in which they were adopted out of the institution, with a particular focus on cognitive and social behavior. In general, the findings suggest that the older a child is at time of adoption (and usually the longer a child has lived in an institution) the lower the child's IQ and poorer the child is with regard to adaptive behavior. Tizard and colleagues, for example, assessed children being raised in residential nurseries in the UK, comparing them to children living with their biological families [66, 67]. Children adopted at a young age had IQ scores that were lower than similarly aged children raised with their biological families, but by age 8 those adopted before age 4.5 were doing better (and on par with children raised in biological families) compared to children adopted later in childhood. Bolstering these early findings are a meta-analysis of IQ among institutionally reared children; here, van IJzendoorn et al. [68] reported that the length of time living in an institution was the best predictor of lower IQ. This finding is somewhat qualified by the context of care in institutions. For example, children adopted from institutions in China appear to fare better than those adopted from Eastern Europe [69]. Country effects likely reflect the level of deprivation children experience in various settings.

Two studies that followed children adopted out of institutions in Romania after the communist Ceausescu era found that recovery of IQ was significantly related to age of adoption. In one study by Ames and Carter [70], those children adopted after 4 months of age displayed lower IQ scores compared to those adopted before 4 months of age; likewise, in the English and Romania Adoptees (ERA) study, children adopted into homes in the UK before six months of age were indistinguishable on IQ compared to controls whereas those adopted after 6 months had IQs that were significantly lower [71].

### 7.1. Social-Emotional Disturbances

Of all the domains studied in previously institutionalized children, the one in which children show the greatest deficits is social and emotional behavior. Goldfarb and colleagues in the late 1940s and 50s described an abnormal psychiatric profile among children and adolescents living in institutions. He described a constellation of behaviors called “over-friendliness” in which children were unable to form deep emotional ties with an adoptive parent [61]. Hodges and Tizard in their studies also found that while IQ scores may have normalized among children with a history of institutionalization, most continued to have seriously disturbed attachments [72]. Here too, some children exhibited persistence of “overly friendly” behavior, whereas others exhibited extremes of social unresponsiveness and emotional inhibition. Similar problems in attachment have been noted in infants raised in Greek [73] and Ukrainian institutions [47], although there, the younger a child was at age of adoption the more likely the child was to have a secure attachment. In the samples studied by Ames and Carter [70] and Rutter et al. (e.g., [74]), there was increased risk for disorganized attachments among young children with a history of institutional rearing.

### 7.2. Psychiatric Disturbances

Children with a history of institutional rearing also have demonstrated significant psychiatric problems. These have ranged from autistic-like social abnormalities [75] to aggressive behaviors and callous unemotional traits [76, 77] to hyperactivity [78] to poor executive control [79–83] and perhaps most characteristic of children with a history of institutional care, inattention/overactivity [67]. Particularly intriguing was Rutter et al.'s [74] observation that nearly 10% of the previously institutionalized sample at age 4 demonstrated socially aberrant behavior they referred to as “quasi-autism.” Although at 4 years, the clinical picture of children so designated was indistinguishable from classic autism, by age 6 years their clinical picture had changed sufficiently that they were designated “quasi” autistic rather than displaying autism proper. By age 11 years, 75% of the children from age 6 continued to manifest quasi-autism, remarkable stability given that autism proper is not due to so-called “maternal deprivation” (cf. [84]). Similar autistic social behaviors have been reported in a small number of children in the Bucharest Early Intervention Project (BEIP; see [85]).

### 7.3. Neural Consequences

Over the past 20 years, there have been a number of studies using different neuroimaging and biological techniques designed to examine the effects of early institutional care on brain and biological development. In terms of effects on the brain, these studies suggest that basic brain structure and function are affected by the experience of early institutionalization. For example, reductions in both gray and white matter volume have been reported [86–88] as have reductions in EEG power [89–91]. Tottenham et al. [92] have reported an enlarged amygdala volume, although this finding has not been replicated by others (cf. [87, 88, 93]). Finally, Gee et al. [94] have examined the functional connectivity between the amygdala and prefrontal cortex in children with a history of institutionalization. They report precocious connectivity in postinstitutionalized children and suggest that this “mature” pattern is a function of adversity and the lack of caregiver buffering early in life.

### 7.4. Biological Effects

In terms of molecular effects, the most striking finding is that children with a history of institutional rearing show reduced telomere length (TL) early in life [95]; more importantly, over the course of the first decade of life such children show a far more dramatic decline in TL than children without a history of institutional care [77]. Accelerated cellular aging may have important implications for subsequent health outcomes.

## 8. Recovery from Early Institutional Rearing

The pernicious effects of institutional rearing on brain development and behavior suggest that deprivation early in life is particularly harmful. This evidence may also inform neuroscience about the presence of critical periods in human development. Studies of young children with a history of institutionalization, in general, cannot address these issues directly since it is not possible to randomize children to contexts of deprivation or family care. Among the most rigorous studies documenting the effects of early institutionalization are the ERA study and the BEIP. The ERA study is a natural experiment following 165 adopted children who had experienced early deprivation in Romanian institutions for varying amounts of time ranging from a few months to 42 months of age and a comparison group of 52 nondeprived adopted Romanian children. Advantaged and motivated adoptive parents provided a dramatic caregiving contrast to the institutional rearing conditions from which ERA study children were adopted. All children were assessed comprehensively at 4, 6, 11, 15, and 22 years of age on measures of cognitive, social, emotional, behavioral, and health outcomes. Investigators documented significant gains in children following adoption (suggesting that the critical periods had remained open, a possible by-product of deprivation), but they identified four deprivation-specific patterns that persisted through all follow-up assessments in some children: severe cognitive impairment, inattention/overactivity, disinhibited attachment (i.e., indiscriminate behavior), and autistic-like social behaviors (i.e., quasi-autism). Associated with these patterns were serious behavioral, emotional, and peer relationship problems extending into adulthood [96]. They also reported that virtually all of the children displaying these deprivation-specific patterns were adopted after the age of 6 months, suggesting that restoring adequate caregiving by 6 months of age led to nearly complete recovery. The children in that study were not adopted at random, so the degree to which these results generalize to nonadopted groups of children who experienced severe early deprivation is unclear.

The BEIP has examined the issue of critical periods in brain and behavioral development in an even more precise fashion. After excluding children with identifiable genetic or neurological syndromes or signs of fetal alcohol exposure, 136 children between six and 31 months of age were recruited from all six institutions for young children in Bucharest. It was assumed that these 136 children would be representative of those placed into Romanian institutions for young children more generally. Following a comprehensive assessment, these 136 children were randomized to care as usual (continued institutional care) or to special foster care that was created, supported, financed, and managed by a BEIP clinical team [97]. Foster care had only recently become legal in Romania and was not widely available at the time the study began. Children were assessed at 30, 42, and 54 months. At that point, the trial was concluded, and the BEIP foster care network was transferred to local governmental authorities. Additional assessments were conducted for all three groups at 8 and 12 years, and another follow-up is underway at age 16 years. (For discussion of the ethical issues the BEIP investigators faced, see [98, 99].)

The design of BEIP allowed examination of the effects of early deprivation on young children with a history of institutionalization, but more importantly for this review, the data are able to address questions about critical periods in exposure to adversity and their effects on brain development and psychological functioning. The 68 children randomized to be taken out of the institutions and placed into foster care ranged in age from 6 to 30 months. Critical periods could be identified by examining their brain and behavioral development at the follow-up assessments as a function of their age of placement into foster families. Findings from BEIP indicate that children placed at or below 24 months of age had higher IQ scores at 54 months of age [100], more mature patterns of brain electrical activity at age 8 [90], more secure attachments to their adult caregivers at 42 months of age [101], less indiscriminate behavior through 8 years [102], and healthier stress responsivity in both sympathetic and cortisol reactivity at 12 years [41]. Not surprisingly, critical periods for recovery varied by domain. For example, for receptive and expressive language, the cut off was placement by 15 months of age [103], whereas for physical growth and stereotypies it was 12 months of age [81, 104].

In addition, some domains of functioning showed intervention effects but no evidence of a critical period. These domains included psychiatric symptoms and disorders [76, 105] and peer social competence [106, 107]. Finally, there were domains of functioning that were mostly unaffected by the intervention (including ADHD; [76, 105]; and most executive functions; [79, 80, 108]) and even a few domains that seemingly were unaffected by exposure to early adversity (face and emotion processing; [79, 96, 109–111]). The lack of critical periods for some domains is not surprising given the complexity and heterogeneity of the domains of functioning being assessed (e.g., psychopathology). We would expect that the more complex the domain of functioning the less likely any one critical period would be identified. Critical periods are reflected in behaviors, but they operate at the level of circuitry [11]. Within broad constructs of clinical interest, such as language, IQ, and attachment, there are multiple critical periods for the different processes underlying language abilities.  Table 1 presents a summary of the findings on critical periods by domain in the BEIP.

The data from the BEIP and the ERA studies provide some of the best evidence for critical periods in brain and behavioral development in the human child. There are continued questions to be raised with regard to exactly how broadly or narrowly shaped these critical periods are and more generally what the timing and dose of exposure is for a particular critical period. However, they clearly identify the importance of family care in the life of the young child. The institutional context documents what is *not* happening in the child-caregiver relationship that affects brain, cognitive, and social development. And the rodent and nonhuman primate data point to the effects of general lack of stimulation and interaction as having a primary influence in these critical period effects.

## 9. Implications and Lessons Learned

Although it is well known that exposure to adverse early experience can derail development (see [112] for a review), the *lack* of experience can be particularly insidious, as the brain awaits instructions to guide its assembly that it fails to receive. As a result, neural circuitry is seriously compromised, which in turn results in delays and impairments in behavior. In the case of institutional care, particularly when children are abandoned in the first months of life and remain in institutional care for more than a few years, the effects are particularly extreme. The evidence we reviewed indicates that recovery from the deleterious effects of institutional care are largely mediated by timing—that is, the age at which a child is removed from an institution and placed into a family. This is also illustrated by the results of the ERA, where children placed into families before six months of age are identical to their nonadoptive siblings, whereas those adopted after six months are at increased risk for persistent trajectories of impaired cognition, disinhibited social behavior, inattention/overactivity, and autistic features.

On the other hand, the results from BEIP are more nuanced. Clear timing effects were apparent at younger ages—those placed into families before 2 years of age fared better than those placed after 2 years of age—but in several domains these timing effects disappeared by the time children were 8 to 12 years old. For example, children randomized to foster care before the age of 24 months had significantly higher IQs at 4.5 years than those randomized after 24 months. However, no timing effects were evident at age 8 and 12 years [107]. Note, however, that we cannot rule out the possibility that the children placed after 24 months started to catch up, whereas the development of those placed before 24 months remained constant. More importantly, however, intervention effects were maintained—at 12 years full scale IQ scores among the children in foster care are still higher than the children who received care as usual, and EEG power remains higher in children placed in foster care than those assigned to the care as usual group.

One possible explanation is compensatory processes in brain development that allow some recovery of function through alternative pathways/neural circuits despite early disturbances in brain architecture. An example is Knudsen's [113] work on visual/auditory mapping in owls, which demonstrated that alterations in input led to new compensatory circuitry. This argues for a sensitive period interpretation of the findings—that is, prolonged and continuous effort (i.e., living in a high-quality foster care family for many years) may overwrite the effects of early deprivation—but only in some domains. Another possibility is that early deprivation temporally extended the sensitive period, making it possible that later placed children continued to accrue benefits compared to the children who experienced care as usual. Better understanding of the development of specific circuits and their sensitivity to environmental input in humans will help clarify these findings.

Millions of orphaned, abandoned, and maltreated children around the world require care outside of their families. Some experience profound neglect while living with their families. Others have parents who seek employment far away and have placed them in less than ideal care settings. The ravages of disease (e.g., HIV AIDS, Ebola, and Zika) and war continue to plague many countries, leading to orphans and sometimes child-headed households. These situations force societies to determine how best to care for orphaned, abandoned, and maltreated children. Evidence we reviewed indicates that the forms of care arranged for such children will play a critical role in their subsequent health and development.

Finally, studies of children experiencing profound neglect have proved particularly informative in elucidating the role of experience, more generally, during critical periods of brain development. This, in turn, has led to new insights into the nature, timing, and duration of the key experiences young children must have to launch them on a pathway of healthy development; they also speak to the importance of intervening early in the lives of children experiencing early neglect (and likely, adversity more generally). We would do well to heed these lessons, as the success of our societies rests on the healthy development of its children, and steps can and should be taken to ensure all children have the opportunity to live up to their developmental potential.

## Figures and Tables

**Table 1:** Timing of placement effects: Domains assessed in the Bucharest Early Intervention Project and and the age before which the intervention had its greatest impact, suggesting a sensitive period for that domain.

Domain assessed	Sensitive period “closes”
Stereotypes	12 months
Expressive language	15 months
Receptive language	15 months
Reading	24 months
Security of attachment	24 months
Organization of attachment	24 months
IQ at 54 months	24 months
ERN during flanker 8 years	20 months
Alpha and theta 8 years	24 months
Teacher-rated social skills 8 years	20 months
Cortisol response 12 years	24 months
RSA response 12 years	18 months
Competence 12 years	20 months
